# Early essential newborn care is associated with increased breastfeeding: a quasi-experimental study from Sichuan Province of Western China

**DOI:** 10.1186/s13006-020-00343-3

**Published:** 2020-11-23

**Authors:** Chen-ran Wang, Xia-yun Li, Lin Zhang, Lin-man Wu, Ling Tan, Fei Yuan, Yao Guo, Sarah Williams, Tao Xu

**Affiliations:** 1 National Center for Women and Children’s Health, Chinese Center for Disease Control and Prevention, Beijing, 100081 China; 2 Beijing Fengtai District Maternal and Child Health Hospital, Beijing, China; 3 Save the Children China Program, Chengdu, China; 4 Sichuan Maternal and Child Health Hospital, Chengdu, China; 5 Mianyang Maternal and Child Health Family Planning Service Center, Mianyang, China; 6 Deyang Jingyang District Family Planning Service Center, Deyang, China; 7 Save the Children, London, EC1M4AR UK

**Keywords:** Early essential newborn care, Breastfeeding, Western China

## Abstract

**Background:**

Breastfeeding is critical to promote maternal and child health. China has set national targets to further improve the exclusive breastfeeding rate. We aimed to examine associations between the provision of early essential newborn care (EENC) and breastfeeding outcomes among full term vaginally delivered neonates in the first 6 months of life.

**Methods:**

We conducted a quasi-experimental study in eight maternal and children’s hospitals in Mianyang City and Deyang City in Sichuan Province of western China. Four hospitals were randomly selected as the intervention group with the implementation of EENC while others as the control group receiving routine care. We assessed effects of EENC on breastfeeding initiation time, duration of first-time breastfeeding, and exclusive breastfeeding rates up to 6 months of age. Data were collected after delivery, at hospital discharge, 1 month, 3 months, and 6 months post birth in the baseline phase from May to June 2017 and post-EENC phase from October to December 2017. We performed univariate analyses to ascertain differences between the two groups, and difference in difference (DID) models to explore the net effects.

**Results:**

Of the 1349 enrolled mother and newborn pairs in our study, 1131 (83.9%) were followed up at 1 month of age, 1075 (79.7%) at 3 months, and 981 (72.7%) at 6 months. EENC was associated with earlier median time to initiate breastfeeding (25 min vs. 33 min, *P* <  0.01), an increased chance of successful first-time breastfeeding (OR 5.53; 95% CI 2.69, 11.40), longer duration of skin to skin contact (SSC) (21.53 min; 95% CI 18.17, 24.89) and longer duration of the first breastfeed (4.16 min; 95% CI 2.10, 6.22), and an increased likelihood of being exclusively breastfed at discharge (74.5% vs. 55.0%, *P* <  0.001), 3 months (OR 3.20; 95% CI 1.01, 10.15), and 6 months (OR 4.91; 95% CI 1.71, 14.13) of age.

**Conclusions:**

EENC enhances breastfeeding initiation and increases exclusive breastfeeding at 6 months of age. Our evidence suggests that nationwide scale up of EENC would increase the exclusive breastfeeding rate in the first 6 months of life.

## Background

Breastfeeding is a highly beneficial and cost-effective public health intervention which has a positive impact on short- and long-term maternal and child health [1–4]. The *Global Strategy for Infant and Young Child Feeding* jointly developed by World Health Organization (WHO) and the United Nations Children’s Fund (UNICEF) recommended that, infants should be exclusively breastfed throughout the first 6 months of life [5]. In 2018, the revised Baby-Friendly Hospital Initiative (BFHI) *Protecting, promoting, and supporting breastfeeding in facilities providing maternity and newborn services* provided the most updated Ten Steps to Successful Breastfeeding; therein Step 4 “*Facilitate immediate and uninterrupted skin-to-skin contact and support mothers to initiate breastfeeding as soon as possible after birth*” is supported by evidence that early initiation of breastfeeding increases the likelihood of a child being exclusively breastfed up to 3–6 months of life [6]. Therefore, identifying interventions that promote early initiation of breastfeeding should be prioritized nationally.

The *National Program of Action Plan for Children Development (2011–2020)* and the *National Nutrition Plan (2017–2030)* launched by the State Council of China both include the target that by 2020, the exclusive breastfeeding rate in the first 6 months of life is expected to increase to 50% [7, 8]. Nevertheless, data on breastfeeding status is inconsistent in important nationwide surveys. The Fifth National Health Service Survey conducted in 2013 showed the exclusive breastfeeding rate for children under 6 months was 58.5% [9], while the *Report on Influential Factors of Breastfeeding in China* issued by China Development Research Foundation in February 2019 demonstrated this rate was only 29.2% and lagged far behind the world’s average level of 43 and 37% in low- and middle-income countries (LMICs) [1, 10]. At present, early breastfeeding initiation rates vary from 18.4% in Pakistan to over 90% in high-income countries such as Australia and Japan, while in China the rate is unknown [11]. The current Chinese situation supports the need to accelerate progress towards early breastfeeding promotion.

Many practices during childbirth threaten early initiation and duration of breastfeeding. These include common practices such as separation of mother and baby too early for measuring weight and length which act as barriers to early mother and newborn contact, and early initiation of breastfeeding [12]. In order to improve these practices, countries in the Asia and Pacific region endorsed the *Action Plan for Healthy Newborn Infants in the Western Pacific Region (2014–2020)* in 2013 [13]. This plan focuses on provision of early essential newborn care (EENC) which contains a package of simple evidence-based interventions to prevent or treat the most important causes of newborn morbidity and mortality. The special attention was paid to improving quality of intrapartum and newborn care during the first 24 h after delivery, including early breastfeeding promotion [13, 14]. Since 2013, eight priority countries with the highest burdens of neonatal mortality in the Asia and Pacific region: Cambodia, China, Lao PDR, Mongolia, Papua New Guinea, the Philippines, Solomon Islands, and Vietnam, have all been supported by WHO to introduce, sustain, and scale-up EENC. Reviews of EENC implementation in these countries showed improvements in newborn care practices and a significant impact on neonatal health outcomes [15, 16]. Compared to the other seven priority countries, China introduced EENC later, with six pilot hospitals in 2016 to over 110 hospitals by the end of 2019 [17]. The National Health Commission (NHC) has been working closely with various partners to optimize the EENC recommendations for a Chinese context, and amends implementation plans to suit local settings [18]. Assessment data of the pilot hospitals in China provided an opportunity to examine the association between EENC interventions and breastfeeding outcomes [19]. More research is needed to identify the most effective ways of implementing EENC in different settings in China, especially in western and rural areas.

Ensuring high-quality newborn healthcare is a core objective of China’s National Plan to achieve the Sustainable Development Goals (SDG) [17]. EENC represents an important strategy to achieve this objective. However, the current childbirth and early newborn care policy and practice guideline in China are not aligned with WHO recommendations for some key interventions [20, 21]. As the Chinese government is taking actions to further improve the breastfeeding situation, more high quality, localized evidence is needed to convince policy makers to change current newborn care regulations. The objective of this study was to explore the associations between the implementation of EENC and breastfeeding, using the pilot data in one of the provinces in western China. The results will help to identify the effects of high-quality EENC intervention on breastfeeding, which can convince policy makers and hospital managers to make changes accordingly.

## Methods

### Study design

This is a quasi-experimental study.

### Settings

This study was conducted in Sichuan Province of western China from May 2017 to December 2017. As one of the pilot hospitals where the implementation of EENC was rolled out successfully since March 2016, Sichuan Provincial Hospital for Women and Children provided qualified faculties and technical support for this study. Mianyang City and Deyang City, with the highest numbers of annual live births in Sichuan Province, were chosen as research settings and randomly assigned as the intervention group (Mianyang City) and the control group (Deyang City). The conditions of medical care services and economic development were equivalent in the two groups. One city level and three county level maternal and children’s hospitals were randomly elected in each group. EENC was introduced in the intervention hospitals while the control hospitals followed routine childbirth and newborn care procedures.

### Participants

#### Inclusion and exclusion criteria

Neonates and their mothers were included in this study according to the following criteria: 1) pregnant women had no history of medical problems and agreed to accept the implementation of EENC with written informed consent; 2) full-term (gestational age: ≥ 37 weeks and < 42 weeks) newborns with birthweight > 2500 g and < 4000 g, who were delivered vaginally. The exclusion criteria included: 1) women with abnormal pregnancy who were admitted to hospitals for any reason, such as those with obstetric emergencies and/or serious underlying diseases; 2) newborns who were admitted to the neonatal unit for any problems. Caesarean deliveries were excluded because the current clinical protocols of caesarean section in China were inconsistent with EENC recommendations and hospitals were not prepared to do so.

Eligible pregnant women were recruited in the prenatal inpatient wards and allocated a number according to order of hospitalization. Those who were included in this study were selected using random number table method. If the women and/or newborns were excluded after delivery, the recruitment and sampling procedure were repeated.

#### Sample size considerations

The sample size of this study was calculated using the following formula (*P*_1_: exclusive breastfeeding rates before hospital discharge in the pre-EENC phase; *P*_2_: exclusive breastfeeding rates before hospital discharge in the post-EENC phase; *P*: $\frac{P_1+{P}_2}{2}$; *Z*_*1*_*-*_*α/2*_/*Z*_*β*_: standard normal deviance at the significance level of α/1-β; *Deff*: design effect):

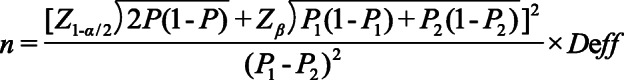



The initial estimate of *P*_*1*_ (46.6%) was applied based on previous publications [22, 23]. We hoped the EENC implementation would increase this rate by 15% and thus made an assumption of *P*_*2*_ (63%). A minimum sample of 255 (*α* = 0.05; *β* = 0.10; *Deff* = 1.2) mother-child pairs per group was therefore calculated with allowance for 10% loss to follow up. The total number of mother-child pairs in the study was 1020 (pre and post*intervention and control = 255*4).

### Implementation of EENC

EENC was introduced in the intervention hospitals in July 2017 by firstly coaching health professionals including obstetricians, obstetric nurses, midwives, pediatrician/ neonatologists, and pediatric nurses using Early Essential Newborn Care Module 2 - *Coaching for the First Embrace - Facilitator’s Guide* [24]*.* The key interventions of EENC include immediate and sustained skin-to-skin (SSC) of mother and newborn for at least 90 min after birth, timely breastfeeding when newborns exhibit feeding cues, delayed umbilical cord clamping, immediate and thorough drying, and neonatal resuscitation for those without spontaneous breathing. Mother and newborn SSC should be the continuous contact that the naked baby is placed against mother’s breast and abdomen until cues of readiness to suck, such as rooting, drooling, tonguing, and biting hands [13, 14, 22]. By September 2017, a quality control assessment was carried out by provincial facilitators to oversee the implementation of EENC and ensure that all trained staffs grasped the skills. The control hospitals implemented routine childbirth care practices. EENC was introduced in the control hospitals after the final data collection.

### Data collection

Pre- and post-intervention data were collected in both groups. The baseline data of the intervention group (*n* = 331) and control group (*n* = 381) were collected from May to June 2017, which was before the EENC introduction. When the intervention hospitals could implement EENC with satisfying quality, we collected post-intervention data in the two groups (*n* = 312 and 325, respectively) from October to December 2017.

**Fig. 1:**
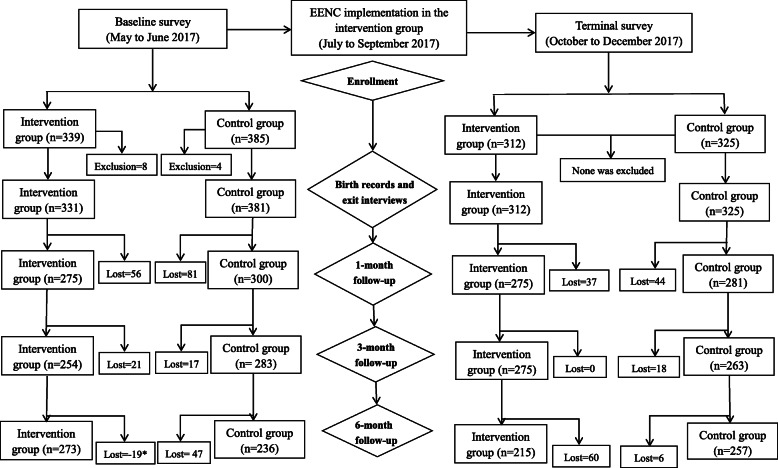
Flow chart for data collection

The same questionnaires were applied in the pre- and post-intervention phase. Birth records on vital signs of mothers and neonates, situation of EENC implementation, and breastfeeding practices (including the time of initiation and duration of breastfeeding), were documented by midwives after delivery. Exit interviews with postpartum mothers were conducted by obstetric nurses in maternity wards to record information about feeding patterns. Defined as Exclusive breastfeeding: infants were fed with only breastfeeding without any liquids or solid food, except for medicine, minerals and vitamin; Artificial feeding: infants were given formula milk only; Breastfeeding: infants received any breastfeeding, combined with formula, prior to discharge. Follow-up data on feeding practices, and mother and child’s health status were collected by child healthcare workers via telephone interviews or home visits at 1, 3, and 6 months post birth. The flowchart for data collection is shown in Fig. 1. This study only focused on the effect of EENC on breastfeeding. The association of EENC with other child health indicators will be published separately.

### Statistical analyses

Data were input into EpiData 3.0 with double-entry method and SPSS statistical software (version 22.0) was used for analyses. All *P* - values were two-sided with the significant difference at 5% level. For continuous variables, data with normal distribution were summarized by mean ± standard deviance (SD), and the statistical differences between the two groups were compared by *t*-tests. Quantitative data with abnormal distribution were demonstrated by median and inter-quartile range (IQR), and Wilcoxon rank sum tests were carried out to ascertain the differences. For categorical variables, data were described by frequencies and proportions, and Pearson chi-squared tests were performed to compare outcomes pre- and post-EENC implementation.

Difference in difference (DID) is a statistical model specific to the nonequivalent design. It is applied to deal with baseline differences between the intervention and control groups and estimate the net effect with control of confounding variables [25–27]. With respect to continuous variables (time of SSC initiation, duration of SSC, duration of the first breastfeeding) as outcomes in this study, general linear model (formula 1) was applied to explain the effect of EENC. Logistic regression model (formula 2) was introduced to examine the intervention effect with categorical variables (successful first-time breastfeeding after SSC, breastfeeding pattern) as outcomes. Estimates of the outcome measures were performed by odds ratio (OR) and corresponding 95% confidence intervals (CI). Effects of EENC on breastfeeding were analyzed by DID with group, time, effect (group*time) as independent variables, and time of SSC initiation, duration of SSC, duration of the first breastfeeding, successful first-time breastfeeding with provision of SSC, and breastfeeding pattern as dependent variables. The definitions of variables are shown in Table 1.

1 $$ Y={\beta}_0+{\beta}_g\times group+{\beta}_t\times time+{\beta}_{effect}\times group\times time+\sum \limits_{j=1}^k{\beta}_j{X}_j+\varepsilon $$

2 $$ \ln \left(\frac{P}{1\hbox{-} P}\right)=\log itP={\beta}_0+{\beta}_g\times group+{\beta}_t\times time+{\beta}_{effect}\times group\times time+\sum \limits_{j=1}^k{\beta}_j{X}_j+\varepsilon $$

**Table 1:** Definition of variables

Variables	Description
*Y*	Dependent variables
Time	Pre-EENC period = 0
	Post-EENC period = 1
Group	Control (Routine care) group = 0
	Intervention (EENC) group = 1
Effect	Group*Time
*X*_*j*_	Covariates
*β*_*0*_	Baseline in the routine care group
*β*_*effect*_	Net effect of the EENC
*β*_*j*_	Regression coefficient of *X*_*j*_
*Ɛ*	Random error

## Results

### Baseline characteristics of study hospitals

In 2016, a total of 7397 live births were registered in the intervention hospitals compared to 7415 live births in the control hospitals. A greater proportion of births were delivered vaginally in the control hospitals (50.1% vs. 36.3%, *χ*^*2*^ = 287.75, *P* <  0.01). As Mianyang City and Deyang City hospitals were the medical centers for maternal near miss, the proportion of caesarean sections was relatively higher than that at the average national level (34.1% in 2016). No statistically significant differences were found in the proportion of medical personnel and their educational level between the two groups (*P* > 0.05).

### Demographic characteristics of study participants

A total of 1349 mother-newborn pairs were recorded in this study, 1131 (83.9%) of whom were followed up at 1 month with a loss rate of 16.1%. 1075 (79.7%) were followed up at 3 months with 20.3% loss and 981 (72.7%) at 6 months with a loss rate of 27.3%. As Table 2 shows, 712 mother-newborn pairs pre-EENC and 637 pairs post-EENC were recorded. Pregnant women enrolled in the study had an average age of 26.3 ± 4.5 years and the mean gestational age was 39.0 ± 1.8 weeks. Newborns enrolled in the study had mean birth length of 49.8 ± 1.3 cm and mean birthweight of 3254.9 ± 370.1 g.

**Table 2:** Demographic characteristics of study participants in the intervention and control hospitals before and after EENC implementation

Variables	Total	Pre-EENC				Post-EENC			
	(*N* = 1349)	Intervention	Control	*t*/*χ*^*2*^	*P*-value	Intervention	Control	*t*/*χ*^*2*^	*P*-value
		(*n* = 331)	(*n* = 381)			(*n* = 312)	(*n* = 325)		
**Maternal**									
Age (Mean ± SD)	26.3 ± 4.5	26.2 ± 4.3	26.5 ± 4.7	−0.82	0.42	26.3 ± 4.4	26.4 ± 4.5	− 0.25	0.80
Education *n* (%)				1.16	0.56			2.57	0.28
Junior middle school	553 (41.0)	144 (43.5)	151 (39.6)			136 (43.6)	122 (37.5)		
High school	416 (30.8)	95 (28.7)	114 (29.9)			98 (31.4)	109 (33.5)		
Higher education	380 (28.2)	92 (27.8)	116 (30.4)			78 (25.0)	94 (28.9)		
Gestational age (Mean ± SD)	39.0 ± 1.8	39.1 ± 2.3	38·9 ± 1.0	0.37	0.71	39.0 ± 2.3	39.0 ± 1.1	0.36	0.72
Gravidity (Mean ± SD)	2.4 ± 1.4	2.4 ± 1.3	2.4 ± 1.4	0.24	0.81	2.5 ± 1.4	2.4 ± 1.4	0.73	0.47
Parity (Mean ± SD)	1.1 ± 0.7	1.0 ± 0.7	1.3 ± 0.7	−5.17	< 0.01	1.1 ± 0.7	1.3 ± 0.8	−3.33	< 0.01
Height, cm (Mean ± SD)	158.8 ± 8.0	158.1 ± 11.5	159.0 ± 5.0	−1.34	0.18	159.0 ± 9.4	159.0 ± 4.4	−0.03	0.98
Weight, kg (Mean ± SD)	66.9 ± 11.1	67.6 ± 11.8	66.8 ± 11.3	0.92	0.36	67.0 ± 9.9	66.4 ± 11.1	0.68	0.50
**Infant**									
Gender *n* (%)				1.33	0.25			0.26	0.61
Male	684 (50.7)	165 (50.0)	207 (54.3)			156 (50.0)	156 (48.0)		
Female	665 (49.3)	166 (50.0)	174 (45.7)			156 (50.0)	169 (52.0)		
Length, cm (Mean ± SD)	49.8 ± 1.3	49.9 ± 1.2	49.6 ± 1.3	3.71	< 0.01	50.0 ± 1.3	49.6 ± 1.3	4.14	< 0.01
Birthweight, g (Mean ± SD)	3256.3 ± 370.3	3274.1 ± 353.6	3243.2 ± 367.4	1.14	0.26	3295.5 ± 410.5	3216.0 ± 345.7	2.92	< 0.01

In the pre-EENC samples, the control group had higher parities (1.0 ± 0.7 vs. 1.3 ± 0.7, *P* <  0.001) and lower birth length (49.9 ± 1.2 cm vs. 49.6 ± 1.3 cm, *P* <  0.01) than the intervention group. In the post-EENC samples, the intervention group had greater birthweight (3295.5 ± 410.5 g vs. 3216.0 ± 345.7 g, *P* <  0.01) than the control group. Maternal age, education, gestational age, height, weight, and neonatal gender were not statistically different in both periods (*P* > 0.05).

### Breastfeeding in the two groups pre- and post-EENC implementation

There were significant between-group baseline imbalances in duration of SSC, duration of the first breastfeeding, time of early breastfeeding initiation, and breastfeeding patterns (*P* <  0.05). The intervention groups had greater duration of SSC and duration of first breastfeeding, while the control group had greater time of early breastfeeding initiation.

After EENC implementation, median time of SSC initiation was considerably shorter from a large range of 10 to 0.7 min in the intervention group whilst in the control group from 6 to 3 minutes. Early breastfeeding initiation time was significantly different between the two groups after EENC had been introduced to the intervention hospitals, with the intervention group initiating breastfeeding mostly within 10–40 min and the control group within 21–54 min (*P* <  0.01). After EENC intervention, 91.1% versus 33.3% of the newborns achieved successful breastfeeding with provision of SSC in the intervention and control groups, respectively. The feeding rate before discharge in the intervention group decreased from 35.5 to 23.2%. Exclusive breastfeeding rates prior to discharge (74.5% vs. 55.0%, *P* <  0.01) and at 6 months (48.5% vs. 35.5%, *P* <  0.01) were significantly different between the two groups (Table 3).

**Table 3:** EENC practices and breastfeeding in the intervention and control hospitals before and after EENC implementation, from birth to 6 months post birth

Characteristics	Pre-EENC				Post-EENC			
	Intervention *n* (*%*)	Control *n* (*%*)	*t*/*z*/*χ*^*2*^	*P-v*alue	Intervention *n* (*%*)	Control *n* (*%*)	*t*/*z*/*χ*^*2*^	*P-v*alue
Time of SSC initiation, min (Median_,_ IQR)	10 (4–21)	6 (4–18)	−1.45	0.15	0.7 (0.3–5.1)	3 (1–7)	5.23	< 0.01
Duration of SSC, min (Median_,_ IQR)	30 (15–32)	0 (0–10)	− 16.94	< 0.01	90 (37–105)	0 (0–24)	−20.08	< 0.01
Time of breastfeeding initiation, min (Median_,_ IQR)	16 (7–32)	37 (24–60)	−11.83	< 0.01	25 (10–40)	33 (21–54)	−6.03	< 0.01
Duration of the first breastfeeding, min (Median_,_ IQR)	30 (20–30)	25 (10–30)	−4.18	< 0.01	35 (30–50)	20 (15–30)	−12.94	< 0.01
Breastfeeding initiation within the first hour			50.09	< 0.01			24.13	< 0.01
Yes	280 (90.3)	240 (67.6)			270 (90.6)	236 (75.6)		
No	30 (9.7)	115 (32.4)			28 (9.4)	76 (24.4)		
Successful first-time breastfeeding with provision of SSC			34.16	< 0.01			146.28	< 0.01
Yes	244 (77.0)	53 (47.3)			278 (91.1)	36 (33.3)		
No	73 (23.0)	59 (52.7)			27 (8.9)	72 (66.7)		
Feeding pattern								
Prior to discharge			13.31	< 0.01			33.27	< 0.01
Exclusive breastfeeding	203 (62.1)	179 (48.5)			228 (74.5)	177 (55.0)		
Mixed feeding	116 (35.5)	181 (49.1)			71 (23.2)	143 (44.4)		
Artificial feeding	8 (2.4)	9 (2.4)			7 (2.3)	2 (0.6)		
1 month post birth			4.03	0.13			1.60	0.45
Exclusive breastfeeding	190 (69.6)	218 (74.7)			208 (75.7)	218 (78.1)		
Mixed feeding	76 (27.8)	62 (21.2)			62 (22.5)	53 (19.0)		
Artificial feeding	7 (2.6)	12 (4.1)			5 (1.8)	8 (2.9)		
3 months post birth			8.52	0.01			17.70	< 0.01
Exclusive breastfeeding	194 (74.0)	194 (71.1)			167 (61.4)	200 (76.0)		
Mixed feeding	61 (23.3)	56 (20.5)			92 (33.8)	47 (17.9)		
Artificial feeding	7 (2.7)	23 (8.4)			13 (4.8)	16 (6.1)		
6 months post birth			10.34	< 0.01			39.39	< 0.01
Exclusive breastfeeding	129 (47.3)	118 (50.0)			117 (48.5)	81 (35.5)		
Mixed feeding	127 (46.5)	86 (36.4)			117 (48.5)	98 (43.0)		
Artificial feeding	17 (6.2)	32 (13.6)			7 (3.0)	49 (21.5)		

### Net effect of EENC on breastfeeding

Due to significant baseline differences between the two groups, DID model was used to evaluate the net effect of EENC. Compared with infants receiving routine care, time of SSC initiation was shortened by 3.55 min (95% CI 0.92, 6.18; *P* <  0.01) in the intervention group. Duration of SSC and duration of the first breastfeeding increased by 21.53 min (95% CI 18.17, 24.89; *P* <  0.01) and 4.16 min (95% CI 2.10, 6.22; *P* <  0.01), respectively (Table 4). Mothers receiving EENC were 5.53 times more likely to ensure the first breastfeeding with provision of SSC (OR 5.53; 95% CI 2.69, 11.40; *P* <  0.01). Implementation of EENC was also associated with an increased rate of exclusive breastfeeding at 3 months (OR 3.20; 95% CI 1.01, 10.14; *P* = 0.05) and 6 months (OR 4.91; 95% CI 1.71, 14.13; *P* < 0.01) of age. Statistically significant difference at 1-month (OR 1.02; *P* > 0.05) post birth was not observed in this study (Table 5).

**Table 4:** Net effect of EENC implementation on SSC and breastfeeding using general linear DID model

Dependent variable	Mode	*β*	*SE*	*t*	*P-v*alue	95% *CI* for *β*	
						Lower bound	Upper bound
Time of SSC initiation	Constant	14.58	0.68	21.46	< 0.01	13.24	15.92
	Time	−7.16	1.30	−5.50	< 0.01	−9.71	4.61
	Effect	−3.55	1.34	−2.65	< 0.01	−6.18	−0.92
Duration of SSC	Constant	6.18	1.17	5.30	< 0.01	3.89	8.46
	Effect	21.53	1.71	12.58	< 0.01	18.17	24.89
Duration of the first breastfeeding	Constant	23.04	0.60	38.30	< 0.01	21.86	24.22
	Effect	4.16	1.05	3.96	< 0.01	2.10	6.22

**Table 5:** Net effect of EENC implementation on breastfeeding using logistic regression DID model

Dependent variable	Mode	*β*	*SE*	*χ*^*2*^	*P-v*alue	*OR*	95% CI for OR	
							Lower bound	Upper bound
Successful first-time breastfeeding after SSC	Constant	−0.11	0.19	0.32	< 0.01			
	Group	1.31	0.23	32.20	< 0.01	3.72	2.36	5.86
	Time	−0.59	0.28	4.43	0.04	0.56	0.32	0.96
	Effect	1.71	0.37	21.54	< 0.01	5.53	2.69	11.40
Exclusive breastfeeding-1 months post birth^a^	Constant	−3.73	0.45	67.86	< 0.01			
	Group	0.40	0.49	0.68	0.41	1.49	0.58	3.87
	Time	0.41	0.47	0.76	0.39	1.50	0.60	3.74
	Effect	0.02	0.76	0.00	0.98	1.02	0.23	4.49
Exclusive breastfeeding-3 months post birth^a^	Constant	2.55	0.29	78.62	< 0.01			
	Group	−1.19	0.44	7.20	0.01	0.30	0.13	0.73
	Time	−0.39	0.34	1.33	0.25	0.68	0.35	1.32
	Effect	1.16	0.59	3.89	0.05	3.20	1.01	10.14
Exclusive breastfeeding-6 months post birth^a^	Constant	1.31	0.20	42.87	< 0.01			
	Group	0.72	0.33	4.90	0.03	2.06	1.09	3.90
	Time	−0.80	0.27	8.88	0.00	0.45	0.26	0.76
	Effect	1.59	0.54	8.73	0.00	4.91	1.71	14.13

^a^Reference: Artificial feeding

## Discussion

The results from our study indicated that EENC was significantly associated with early breastfeeding initiation. Mothers who received SSC in EENC implementation experienced shorter median time to initiate breastfeeding (25 min vs. 33 min) and were more likely to ensure first-time breastfeeding (OR 5.53). Similar findings have been reported in previous studies carried out by Conroy et al. in the USA, Marmood et al. in Pakistan, and Safari et al. in Iraq [28–30]. This could be explained by a strong biological mechanism. Shorter time to initiate early breastfeeding can be attributed to EENC through immediate SSC between the mother and her newborn. In the first couple of hours after birth, if the newborn experiences uninterrupted SSC with the mother immediately after birth, it makes it easier for him/her to crawl towards the mother’s nipple, thus effectively contributing to initiate breastfeeding activity [31–33]. Therefore, the first 2 hours after birth, which is considered a “sensitive period” for maternal odors, tactility, and temperature, is the optimum time for infants to initiate breastfeeding. However, the newborns receiving routine care commonly have no chance of receiving immediate SSC because they are separated from their mother. Additionally, SSC is often interpreted by a set of outdated and harmful practices so the first successful breastfeeding is delayed [31, 34]. The effect that EENC decreased initiation time of SSC and breastfeeding found in this study was consistent with immediate postnatal care advocated by WHO and UNICEF in the updated BFHI [5, 6].

Our analysis also suggested that prolonged duration of SSC (21.53 min) and first-time breastfeeding (4.16 min) occurred concomitant with the introduction of EENC. Longer duration of the first breastfeed in mothers receiving EENC results from sustained mother and newborn SSC, by which increased breast milk production prolongs duration of first-time breastfeeding [22], and then enhanced maternal satisfaction may occur [28–30]. These findings favored WHO’s recommendation that “SSC should begin ideally at birth and last continually until the end of the first breastfeeding” [13, 34]. Under the leadership of NHC, Chinese baby-friendly hospitals’ standards were reviewed since June in 2014 and revised based on guidelines updated by WHO and UNICEF in 2018 [8], with the aim to provide high-quality maternal and newborn services. EENC facilitates the creation of an enabling environment where maternal and newborn health is prioritized.

In the present study, the implementation of EENC was associated with increased exclusive breastfeeding rates during the early postpartum period and at 1 to 6 months of age. The associations were significant among infants at discharge (74.5% vs. 55.0%), 3 months (OR 3.20), and 6 months (OR 4.91) of age. Our findings were consistent with previous researches, which showed that infants provided with SSC were more likely to be breastfed at hospital discharge [33], day 28 [32], 1 month [29], and 6 weeks after birth [35]. A review by Moore et al. in 2016 of 38 trials with 3472 pairs covering 21 countries found that women who received SSC were more likely to exclusively breastfeed their babies from 6 weeks to 6 months (RR 1.50; 95% CI 1.18, 1.90) of age [36]. Increased exclusive breastfeeding rates in the intervention group were noted for mothers after cesarean section as well [36, 37]. The relationship between SSC and increased exclusive breastfeeding rates was partly explained by a dose-response association observed by Bramson et al. [38]. But there was no evident effect on exclusive breastfeeding rates at 4 months between the two groups in the study conducted by Carfoot et al. in the north of England, similarly our study did not observe a statistically significant difference at 1 month post birth [22]. This inconsistency in the long-term effect of SSC on breastfeeding may be attributed to inconsistent time of SSC initiation and duration in various studies, inconstant willingness of further compliance influenced by maternal knowledge of EENC [20], different breastfeeding assessment tools [36], babies’ ability to suck [6], and postpartum status including maternal satisfaction [29], nipple proctractility and routine breastfeeding guidance accessible to mothers after delivery and during lactation [31].

### Limitations

The present study has some limitations. The sample size was calculated based on exclusive breastfeeding rates before hospital discharge, for which reason the statistical test power of EENC on breastfeeding at follow up is low. Hence future studies will require a larger number of mother-newborn pairs for a higher degree of precision and establishment of a possible dose-response association [36]. Other confounding factors that might influence breastfeeding such as the intention to breastfeed, lactation difficulties, and attendance at support groups were not collected. With regard to baseline differences between the intervention and control groups, we applied DID models to control the nonequivalence, but statistical methods are not available to completely eliminate differences in baseline information. Our study was conducted in Sichuan Province of western China, and the results cannot be generalized to China as a whole. Furthermore, selection bias, information bias, and the Hawthorne effect may occur in the study since blinding and concealed random allocation were not available to this study.

## Conclusions

As the first experimental study investigating effects of early essential newborn care implementation on breastfeeding in China, our findings show that EENC can promote the early initiation of breastfeeding in the delivery room and exclusive breastfeeding rates in the early stages of life. We recommend that hospital managers and policy makers scale up early essential newborn care in light of our evidence and the corroborating evidence of other referenced studies.

## Data Availability

The datasets used in the study are available from the corresponding author upon reasonable request.

## Abbreviations

CI: Confidence intervals
BFHI: Baby-Friendly Hospital Initiative
DID: Difference in difference
EENC: Early essential newborn care
IQR: Inter-quartile range
KMC: Kangaroo mother care
LMICs: Low- and middle-income countries
NHC: National Health Commission
OR: Odds ratio
SD: Standard deviance
SDG: Sustainable Development Goals
SSC: Skin-to-skin contact
UNICEF: United Nations Children’s Fund
WHO: World Health Organization
